# Development of TiO_2_-coated YSZ/silica nanofiber membranes with excellent photocatalytic degradation ability for water purification

**DOI:** 10.1038/s41598-020-74637-1

**Published:** 2020-10-20

**Authors:** Jin Young Huh, Jongman Lee, Syed Zaighum Abbas Bukhari, Jang-Hoon Ha, In-Hyuck Song

**Affiliations:** 1 Powder & Ceramics Division, Korea Institute of Materials Science (KIMS), 797 Changwondaero, Seongsangu, Changwon, 51508 Republic of Korea; 2 Department of Advanced Materials Engineering, University of Science & Technology (UST), 797 Changwondaero, Seongsangu, Changwon, 51508 Republic of Korea

**Keywords:** Engineering, Materials science

## Abstract

Numerous reports have elucidated that TiO_2_ nanoparticles (TiO_2_-NPs) exhibit respectable photocatalytic degradation capacities due to their high specific surface areas. However, the current recovery process leads to a loss of TiO_2_-NPs; therefore, there is a need to immobilize TiO_2_-NPs on the substrate used. Herein, TiO_2_-coated yttria-stabilized zirconia/silica nanofiber (TiO_2_-coated YSZ/silica NF) was prepared by coating TiO_2_ on the surface of YSZ/silica NF using a sol–gel process. The TiO_2_ coating layer on the nanofiber surface improved the separation ability of the membrane as well as the photocatalytic degradation ability. The pore size of the TiO_2_-coated YSZ/silica NF membrane was less than that of the pristine YSZ/silica NF membrane, and it rejected over 99.6% of the 0.5 μm polymeric particles. In addition, the TiO_2_-coated YSZ/silica NF membrane showed excellent adsorption/degradation of humic acid (HA, 88.2%), methylene blue (MB, 92.4%), and tetracycline (TC, 99.5%). Six recycling tests were performed to evaluate the reusability of the TiO_2_-coated YSZ/silica NF membrane. The adsorption/degradation efficiency for HA, MB, and TC decreased by 3.7%, 2.8%, and 2.2%, respectively. We thus verified the high separation ability, excellent photocatalytic degradation ability, and excellent reusability of the TiO_2_-coated YSZ/silica NF membranes.

## Introduction

Electrospinning technology can continuously produce fibers with diameters that range from several nanometers to several micrometers by using a strong electric field. Electrospinning technology has numerous advantages including the provision of (1) a wide selection of materials, (2) a controllable pore structure, (3) high interconnectivity, and (4) the fabrication of complex structures^1–3^. For these reasons, electrospun nanofibers have been applied in various environmental fields such as (1) water and air purification membranes (or filters)^4–6^, (2) photocatalysts^7^, (3) adsorption^8^, and (4) gas sensors^9^. In addition, electrospun nanofibers have been the focus of various studies aimed at the simple manufacture of polymer materials. However, polymer nanofibers cannot be used under harsh conditions due to their (1) poor thermal stability, (2) low chemical durability. To overcome this problem, numerous researchers have developed inorganic nanofibers (such as ceramics) using electrospinning technology^10–14^.

In recent decades, the use of photocatalytic nanoparticles (NPs) has been considered as an excellent water purification technology^15–17^. This is because water purification technology using photocatalytic NPs uses light energy, rendering it an eco-friendly technology. Further, photocatalytic degradation processes do not produce harmful substances. In particular, TiO_2_ is a representative photocatalyst that is widely applied for environmental purification purposes since it has various advantages such as (1) strong optical adsorption, (2) chemical stability, and (3) low cost. Moreover, TiO_2_ NPs have good photocatalytic degradation capacities due to their high specific surface areas. However, there are several challenges faced including difficulties in separating, recovering, and recycling TiO_2_-NPs. Moreover, TiO_2_-NPs easily form agglomerates due to their high surface energy. This problem requires additional processes such as centrifugation and filtering in the recovery process of TiO_2_-NPs^18–20^ wherein some TiO_2_-NPs are lost. Ultimately, the loss of TiO_2_-NPs causes secondary water pollution, low reproducibility, and reduced photocatalytic degradation efficiency^21,22^. To address these disadvantages, various studies have been conducted where TiO_2_-NPs are immobilized on various substrates (membranes or filters)^23–28^.

We therefore endeavored to immobilize the TiO_2_ coating layer on electrospun ceramic nanofibers. Several inorganic materials (especially ceramics) were feasible to avoid the risk of polymeric decomposition by OH radicals and hydrogen peroxide (H_2_O_2_) that are generated during the photocatalytic reaction^29–31^. The immobilization of TiO_2_ coating layers on ceramic NF membranes not only addresses the disadvantages of water purification technology using TiO_2_-NPs, but also presents a new direction towards advancing electrospun ceramic NF. Herein, the TiO_2_-coated YSZ/silica NF membrane was developed for three purposes: (1) simultaneous separation and photocatalysis, (2) improved photocatalysis (by large surface area), and (3) self-cleaning capability (by photocatalytic degradation). The achievement of these properties requires not only the improvement of both separation and photocatalysis, but also the prevention of TiO_2_ loss, which is responsible for the reduction of the photocatalytic efficiency. Accordingly, the morphology, mean/largest pore size, and the specific surface area of the TiO_2_-coated YSZ/silica NF membrane were characterized. Furthermore, the gas and pure water permeabilities as well as the rejection rate (%) were analyzed. Lastly, the efficiency of adsorption/photocatalytic degradation was evaluated using major pollutants which represent natural organic matter (HA), organic dye (MB), and antibiotic (TC).

## Results and discussion

### Characterization of TiO_2_-coated YSZ/silica NF

**Figure 1:**
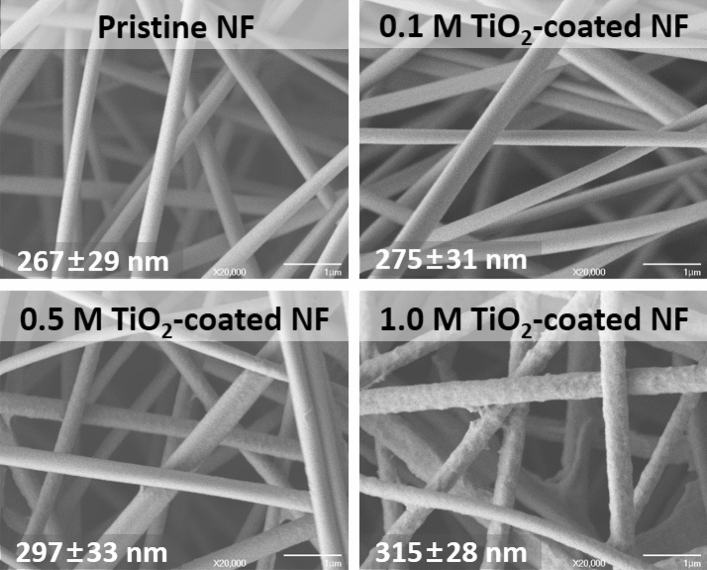
FE-SEM micrographs of YSZ/silica and TiO_2_-coated YSZ/silica NF: pristine NF, 0.1 M TiO_2_-coated NF, 0.5 M TiO_2_-coated NF and 1.0 M TiO_2_-coated NF.

Figure 1 shows the field-emission scanning electron microscopy (FE-SEM) micrographs of pristine YSZ/silica NF and the TiO_2_-coated YSZ/silica NF with various TTIP concentrations. The FE-SEM micrographs show the change in the diameter (TiO_2_ coating layer plus YSZ/silica NF diameter) depending on the TTIP concentration. This indicates that the TiO_2_ coating layer increased as the TTIP concentration raised. For example, the diameters of the YSZ/silica NF and the 0.1 M and 1.0 M TiO_2_-coated YSZ/silica NFs were 267 ± 29 nm, 275 ± 31 nm, and 315 ± 28 nm, respectively. The diameter of 1.0 M TiO_2_-coated YSZ/silica NF increased by approximately 48 nm (thickness of 24 nm) as compared to that of pristine YSZ/silica NF. An increase in diameter of the TiO_2_-coated YSZ/silica NF is able to affect all subsequent membrane characteristics. For example, a reduction in the membrane pore size leads to an increase in the rejection of pollutants, thereby improving the separation capability. The increased TiO_2_ coating layer enhances the photocatalytic degradation efficiency. The formation of a TiO_2_ coating layer on electrospun nanofibers have also been reported using electrospraying^32^ or the sol–gel method^33^. The TiO_2_ coating using electrospraying has a disadvantage in that it resulted in TiO_2_-NP aggregation which over-coated the electrospun nanofibers.

**Figure 2:**
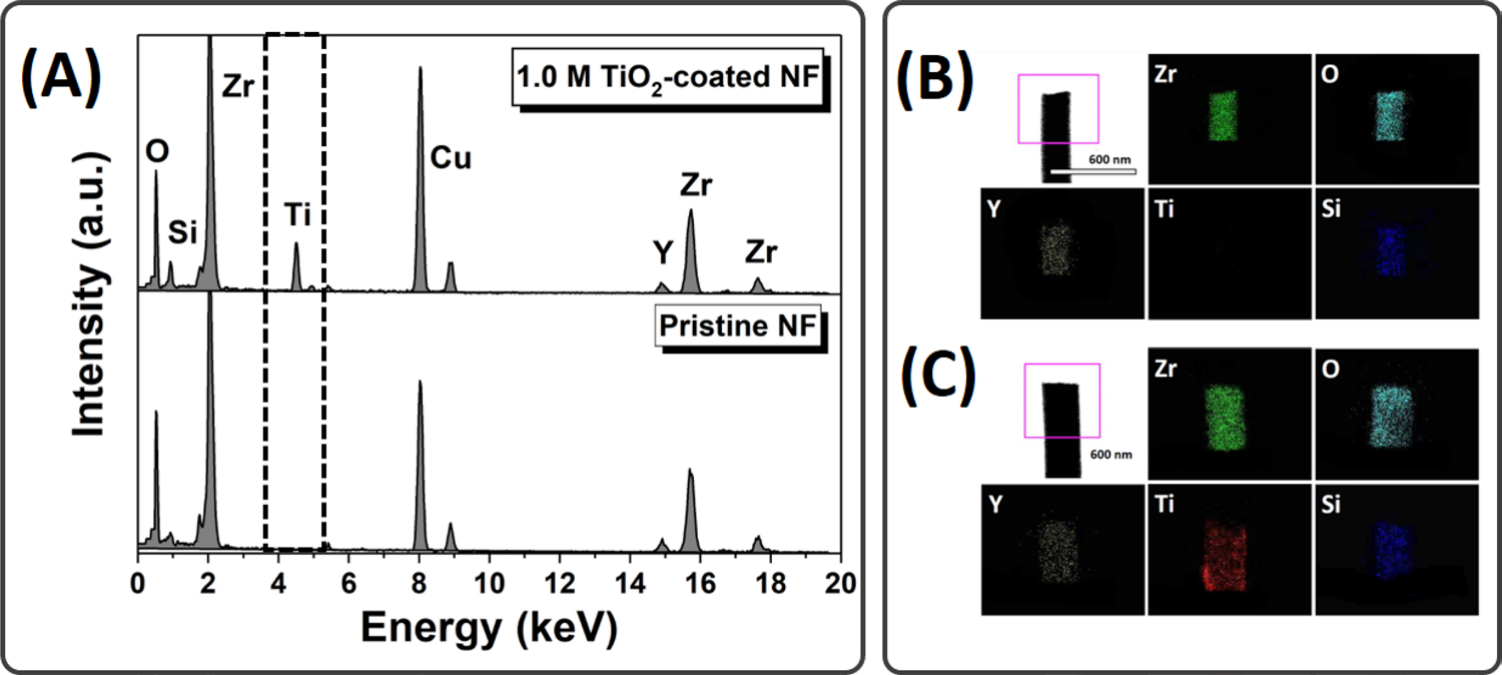
HR-TEM micrographs of YSZ/silica and TiO_2_-coated YSZ/silica NF: (**A**) EDS spectra of pristine NF and 1.0 M TiO_2_-coated NF and elemental maps of (**B**) pristine NF and (**C**) 1.0 M TiO_2_-coated NF.

High-resolution transmission electron microscope (HR-TEM) micrographs and Energy dispersive spectrometry (EDS) spectra confirmed the formation of the TiO_2_ coating on YSZ/silica NF. Figure 2A shows the EDS analysis of YSZ/silica NF and 1.0 M TiO_2_-coated YSZ/silica NF, confirming the co-existence of zirconium, yttrium, silicon, and oxygen. Titanium was only observed in the 1.0 M TiO_2_-coated YSZ/silica NF, indicating that the TiO_2_ coating layer was formed. Similarly, Fig. 2B,C show the elemental maps of YSZ/silica NF and 1.0 M TiO_2_-coated YSZ/silica NF, respectively. It shows a uniform Ti distribution in the 1.0 M TiO_2_-coated YSZ/silica NF, indicating that TiO_2_ was homogeneously coated over the entire nanofiber bundle without aggregation.

**Figure 3:**
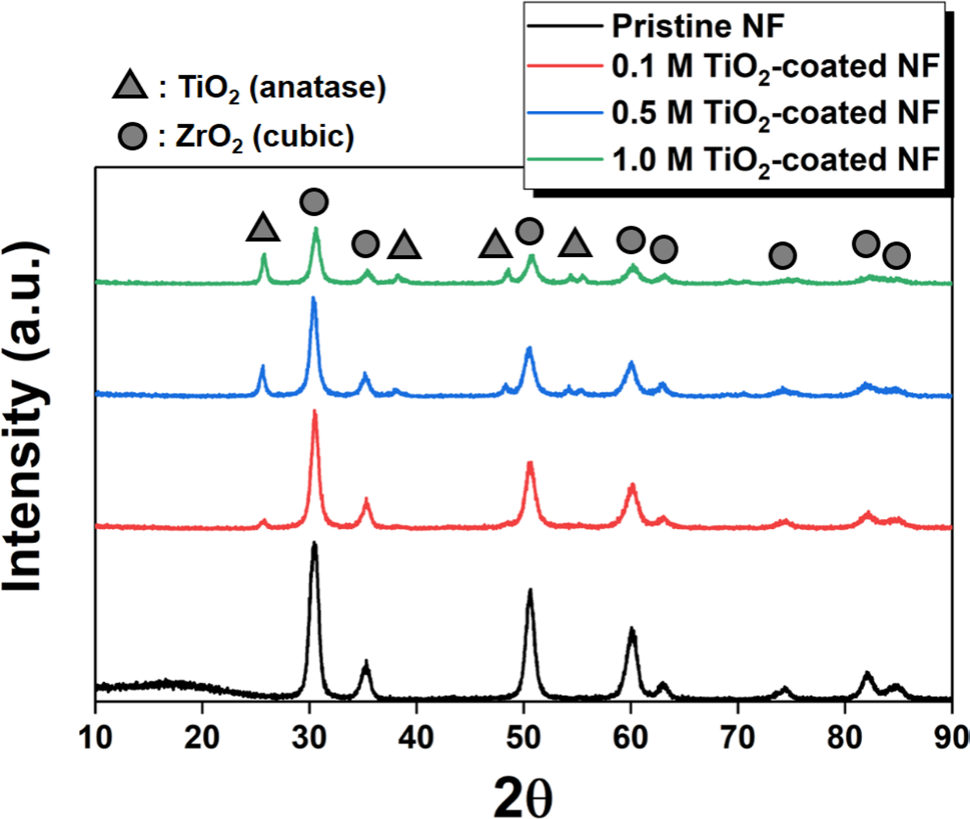
XRD diffractogram of YSZ/silica (pristine) and TiO_2_-coated YSZ/silica NF (0.1, 0.5 and 1.0 M TiO_2_-coated NF).

Figure 3 shows the X-ray diffraction (XRD) diffractogram of both calcined YSZ/silica NF and TiO_2_-coated YSZ/silica NF. The diffractogram of the calcined YSZ/silica NF indicates that the stabilized zirconia had a cubic phase (JCPDS No. 01-081-1550). The peaks assigned to zirconia are 30.0°, 34.7° 50.0°, 59.4°, 62.3°, and 73.4° corresponding to the (111), (200), (220), (311), (222), and (400) crystallographic planes, respectively^11,12^. However, new peaks were observed at 25.3°, 37.8°, 53.9°, and 55.1° in TiO_2_-coated YSZ/silica NF, which show the crystalline anatase TiO_2_ phase (JCPDS No. 89-4320) and crystallographic planes of (101), (004), (105), and (211), respectively^34–36^. In addition, the grain size of the anatase TiO_2_ phase in the 0.1 M, 0.5 M, and 1.0 M TiO_2_-coated YSZ/silica NF was calculated using Scherrer’s equation (16.4 nm, 18.1 nm, and 19.7 nm, respectively). In general, it is known that the grain size of TiO_2_ increases with increasing TTIP concentration^37^. This is because the higher TTIP concentration, the faster the growth rate of nuclei than the nucleation rate^38,39^.

**Figure 4:**
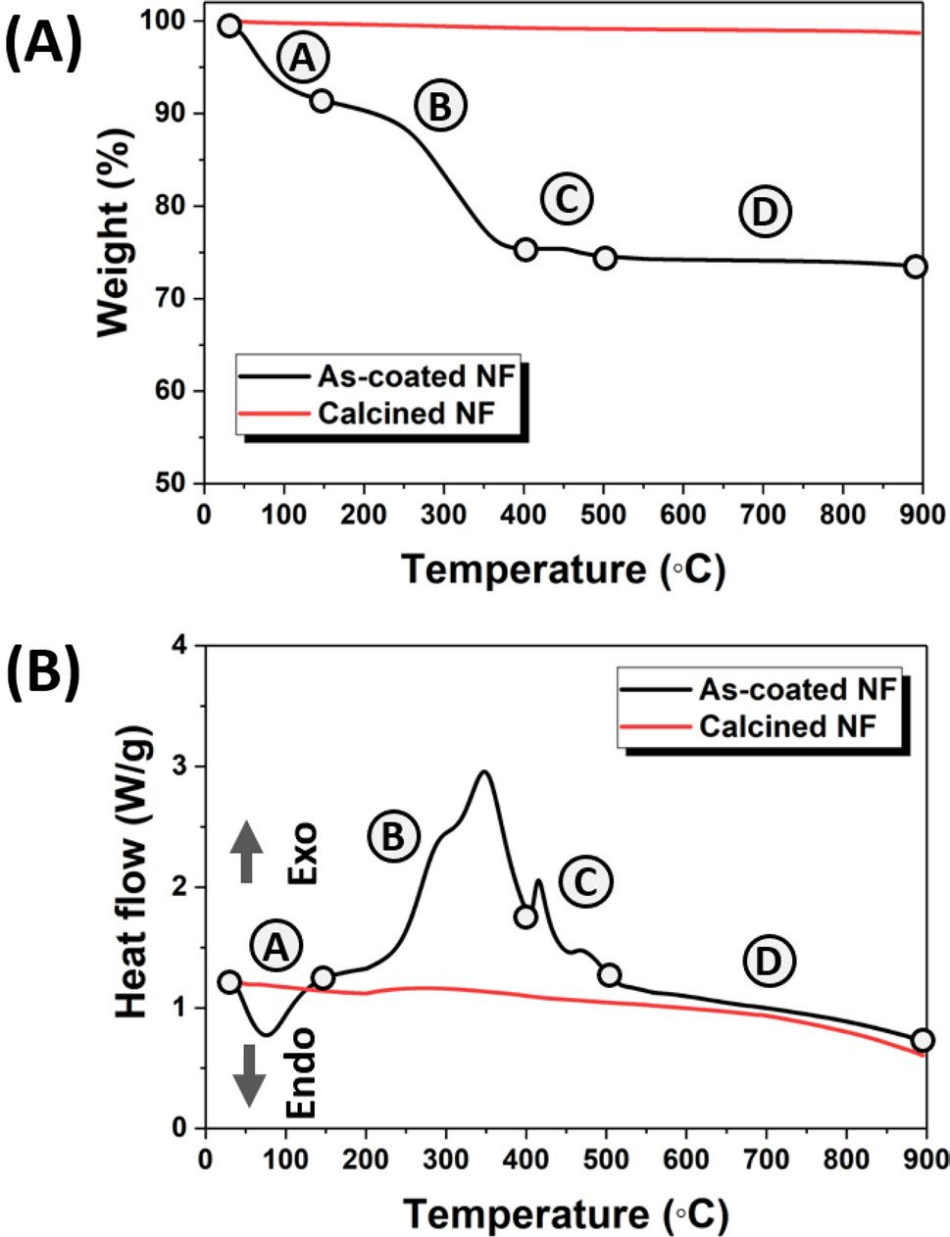
TGA–DSC measurement of as-coated (black line) and calcined (red line) 1.0 M TiO_2_-coated NF: (**A**) TGA and (**B**) DSC.

Thermal gravimetric analysis (TGA) and differential scanning calorimetry (DSC) were conducted on the as-coated and calcined 1.0 M TiO_2_-coated YSZ/silica NF in order to analyze the weight loss and changes in heat capacity. The pyrolysis of the as-coated 1.0 M TiO_2_-coated YSZ/silica NF can be divided into 4 stages, as shown in Fig. 4; Stage A (25–150 ℃), Stage B (150–400 ℃), Stage C (400–500 ℃), and Stage D (500–900 ℃). The TGA curve shows the weight loss of the as-coated 1.0 M TiO_2_-coated YSZ/silica NF in Fig. 4A. Stage A showed a weight loss of approximately 9%, which was attributed to the evaporation of water and pyrolysis of residual organic solvents^40,41^. Stage B showed a weight loss of approximately 16% due to the thermal decomposition of the organic residues that occurred during the oxidation of TTIP that transformed it into an amorphous structure^42,43^. In stage C, the weight was reduced by approximately 2% due to the thermal decomposition of organics present inside the 1.0 M TiO_2_-coated YSZ/silica NF^44,45^. Stage D shows complete removal of all organic compounds and residues, and as a result, no weight change was observed. We analyzed the calcined 1.0 M TiO_2_-coated YSZ/silica NF to identify any remaining organic compounds (for reference). No weight loss was observed for the calcined 1.0 M TiO_2_-coated YSZ/silica NF in the TGA curve.

The DSC curve shows the heat flow of the as-coated 1.0 M TiO_2_-coated YSZ/silica NF, as shown in Fig. 4B. The endothermal peak (76 ℃, in stage A) was due to the evaporation of water, the exothermic peak (350 ℃, in stage B) was caused by the thermal decomposition of organic residue generated during the oxidation of TTIP for transformation into the amorphous structure^46,47^. As shown in stage C, the exothermic peak (417 ℃) is located in the crystallization zone of the anatase TiO_2_ phase^48^. The exothermic peak (468 ℃, in stage C) is due to the thermal decomposition of organic compounds present in the 1.0 M TiO_2_-coated YSZ/silica NF. The calcined 1.0 M TiO_2_-coated YSZ/silica NF showed no change in heat flow as observed in the DSC curve, and this confirmed that no organic compounds were remaining in the sample. If TiO_2_-coated YSZ/silica NF is calcined above 500 ℃, the anatase phase can be converted to the rutile or brookite phase^49^. Studies have shown that the anatase phase has better photocatalytic degradation efficiency than the rutile and brookite phases^50–52^. Thus, in our present study, we calcined the samples at 500 ℃ to obtain the anatase TiO_2_ phase, which has less recombination potential than the rutile and brookite phases.

Table 1 shows the BET analysis results of YSZ/silica NF and TiO_2_-coated YSZ/silica NF. We confirmed the change in the specific surface area (SSA) owing to the formation of the TiO_2_ coating layer on YSZ/silica NF. As shown in Table 1, the SSA of YSZ/silica NF is 9.7 m^2^/g, and the SSA of TiO_2_-coated YSZ/silica NF increased as the TiO_2_ coating layer raised from 0.1 M (10.2 m^2^/g) to 0.5 M (11.6 m^2^/g) and then to 1.0 M (11.7 m^2^/g). This is because the increased TiO_2_ coating layer on the YSZ/silica NF contributes to expand the SSA. The SSA is a critical factor in adsorption and photocatalysis. The SSA of electrospun ceramic nanofiber membranes have smaller than NPs, but higher than that of conventional ceramic membranes [especially microfiltration (MF) and ultrafiltration (UF) membranes]^53,54^. TiO_2_-NPs generally have a high SSA, but the problems of aggregation, separation, and recovery hinder their applicability. Although the SSA of TiO_2_-coated YSZ/silica NF is relatively smaller than that of the TiO_2_-NPs, our research was able to compensate for the drawbacks of TiO_2_-NPs.

**Table 1:** SSA of YSZ/silica (pristine) and TiO_2_-coated YSZ/silica NF.

	Pristine NF	0.1 M TiO_2_-coated NF	0.5 M TiO_2_-coated NF	1.0 M TiO_2_-coated NF
SSA (m^2^/g)	9.7	10.2	11.6	11.7

**Figure 5:**
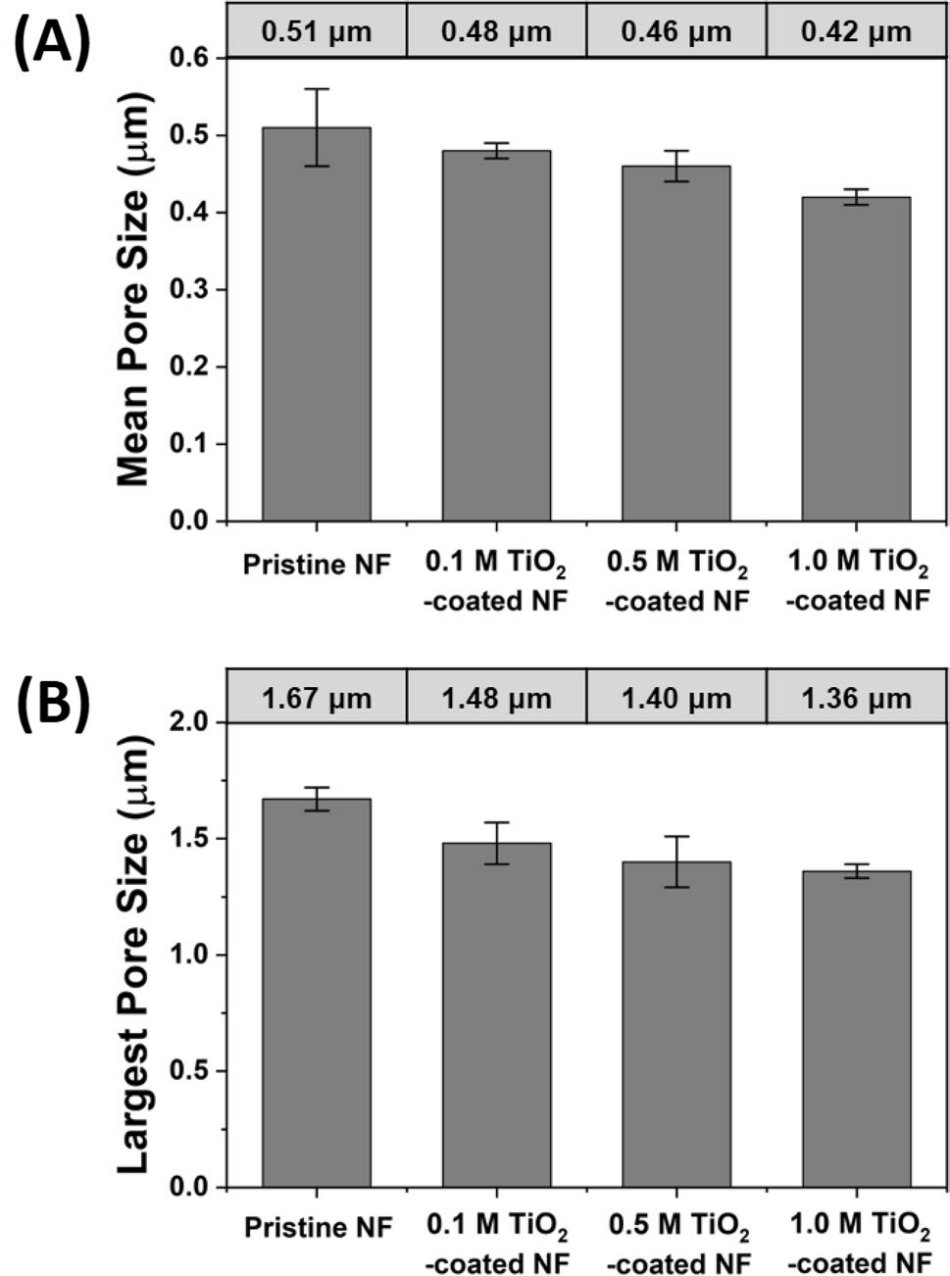
CFP of YSZ/silica (pristine) and TiO_2_-coated YSZ/silica NF. (**A**) The mean pore size and (**B**) the largest pore sizes were measured at the concentration of TiO_2_ (0, 0.1, 0.5, and 1.0 M).

Figure 5 shows the mean and largest pore size of YSZ/silica NF and TiO_2_-coated YSZ/silica NF membranes through the analysis of capillary flow porometry (CFP). The mean pore size of the YSZ/silica NF membrane (0.51 ± 0.05 μm) gradually decreased as the TTIP concentrations increased from 0.1 M (0.48 ± 0.01 μm) to 0.5 M (0.46 ± 0.02 μm) and then to 1.0 M (0.42 ± 0.01 μm) (in Fig. 5A). The largest pore size of the YSZ/silica NF membrane (1.67 ± 0.05 μm) proportionally decreased as the TTIP concentrations increased from 0.1 M (1.48 ± 0.09 μm) to 0.5 M (1.40 ± 0.11 μm) and then to 1.0 M (1.36 ± 0.03 μm) (in Fig. 5B). The mean and large pore size of the TiO_2_-coated YSZ/silica NF membranes decreased with increasing TTIP concentration due to the increasing thickness of the nanofibers, which decrease the pore size of the membrane. This reduction in pore size affects the membrane performance.

### Membrane performance of TiO_2_-coated YSZ/silica NF

Fig. S1 shows the result of gas and pure water permeability for the largest pore size of YSZ/silica NF and TiO_2_-coated YSZ/silica NF membranes. In general, gas and pure water permeability increase as the largest pore size of the membrane rises^55,56^. The largest pore size determines the gas and pure water permeability because the fluid flows to the region with least resistance to water mobility corresponding to the largest pore size, which thus determines the gas and pure water permeability^14^. In this study, as shown in Fig. S1, the largest pore size decreases as the TiO_2_ coating layer increases. Figure S1(a) shows the gas permeability of YSZ/silica NF membrane (largest pore size: 1.67 μm) is 23.4 ± 1.6 L/cm^2^ min bar, and the gas permeability of 1.0 M TiO_2_-coated YSZ/silica NF membrane (largest pore size: 1.36 μm) is 11.8 ± 0.9 L/cm^2^ min bar. In addition, the pure water permeability of YSZ/silica NF membrane is 46,704 ± 2064 L/m^2^ h bar and the pure water permeability of 1.0 M TiO_2_-coated YSZ/silica NF membrane is 31,992 ± 3766 L/m^2^ h bar, as shown in Fig. S1(b). Thus, the gas and pure water permeability of TiO_2_-coated YSZ/silica NF membranes decrease as the largest pore size reduces. Nevertheless, conventional multi-layer ceramic membranes (especially MF and UF membranes) have a large number of closed pores, which decreases the gas and pure water permeability relative to electrospun ceramic nanofiber membranes that have an open pore structure^14,57^. For example, the gas permeability of ceramic membranes is approximately 0.001 L/cm^2^ min to 0.004 L/cm^2^ min, and the pure water permeability is approximately 250 L/m^2^ h to 600 L/m^2^ h^58–60^. Therefore, the gas and pure water permeability of the TiO_2_-coated YSZ/silica NF membranes is much higher than that of ceramic membranes.

**Figure 6:**
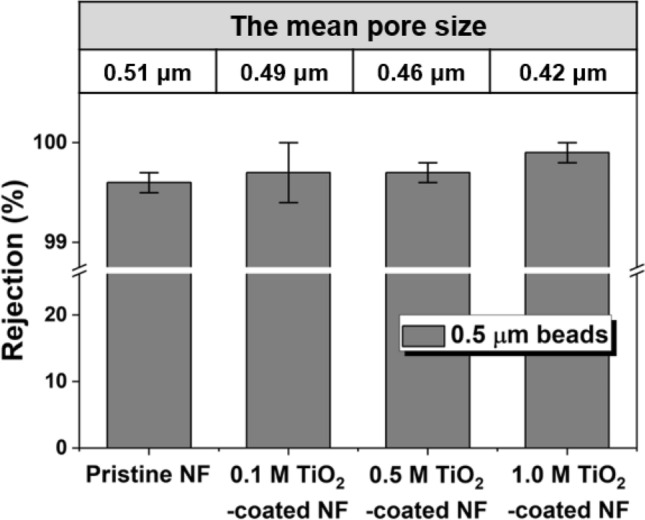
The rejection rate (%) of YSZ/silica (pristine) and TiO_2_-coated YSZ/silica NF with 0.5 μm polymeric beads.

Figure 6 shows the rejection rate (%) of YSZ/silica NF and TiO_2_-coated YSZ/silica NF membranes using 0.5 μm polymeric particles. The mean pore size of YSZ/silica NF and TiO_2_-coated YSZ/silica NF membranes affected the rejection rate (%) because the mean pore size corresponds to the average index of the pores of the membrane can separate particles larger than the mean pore size. The YSZ/silica NF membrane with the largest mean pore size (0.51 μm) showed the lowest rejection rate (99.6% ± 0.1%), and the 1.0 M TiO_2_-coated YSZ/silica NF membrane with the smallest mean pore size (0.42 μm) showed the highest rejection rate (99.9% ± 0.1%). Overall, we confirmed an excellent rejection rate (more than 99.6%) in all the membranes. The difference in the rejection rates (%) is due to the mean pore size of TiO_2_-coated YSZ/silica NF membranes, which was mostly less than 0.5 μm as shown in Fig. 5A. However, the mean pore size of the YSZ/silica NF membrane was 0.51 μm, which was almost equal to or larger than the diameter of the polymeric particles (0.5 μm), but it was measured at a high rejection rate (%) of approximately 99.6% ± 0.1%. This is observed because the structure of the YSZ/silica NF membrane is so intricately entangled into several layers that the polymeric particles undergo the tortuous pathway through the filtration process^14^. The variation in the rejection rate (%) is directly correlated to a difference in the mean pore size of the TiO_2_-coated YSZ/silica NF membranes, which gradually decreased due to the increase in the TiO_2_ coating layer. In other words, the post-processing for the formation of TiO_2_ coating layer on YSZ/silica NF is able to govern the pore size and rejection rate (%).

### Photocatalytic degradation of the TiO_2_-coated YSZ/silica NF membrane

**Figure 7:**
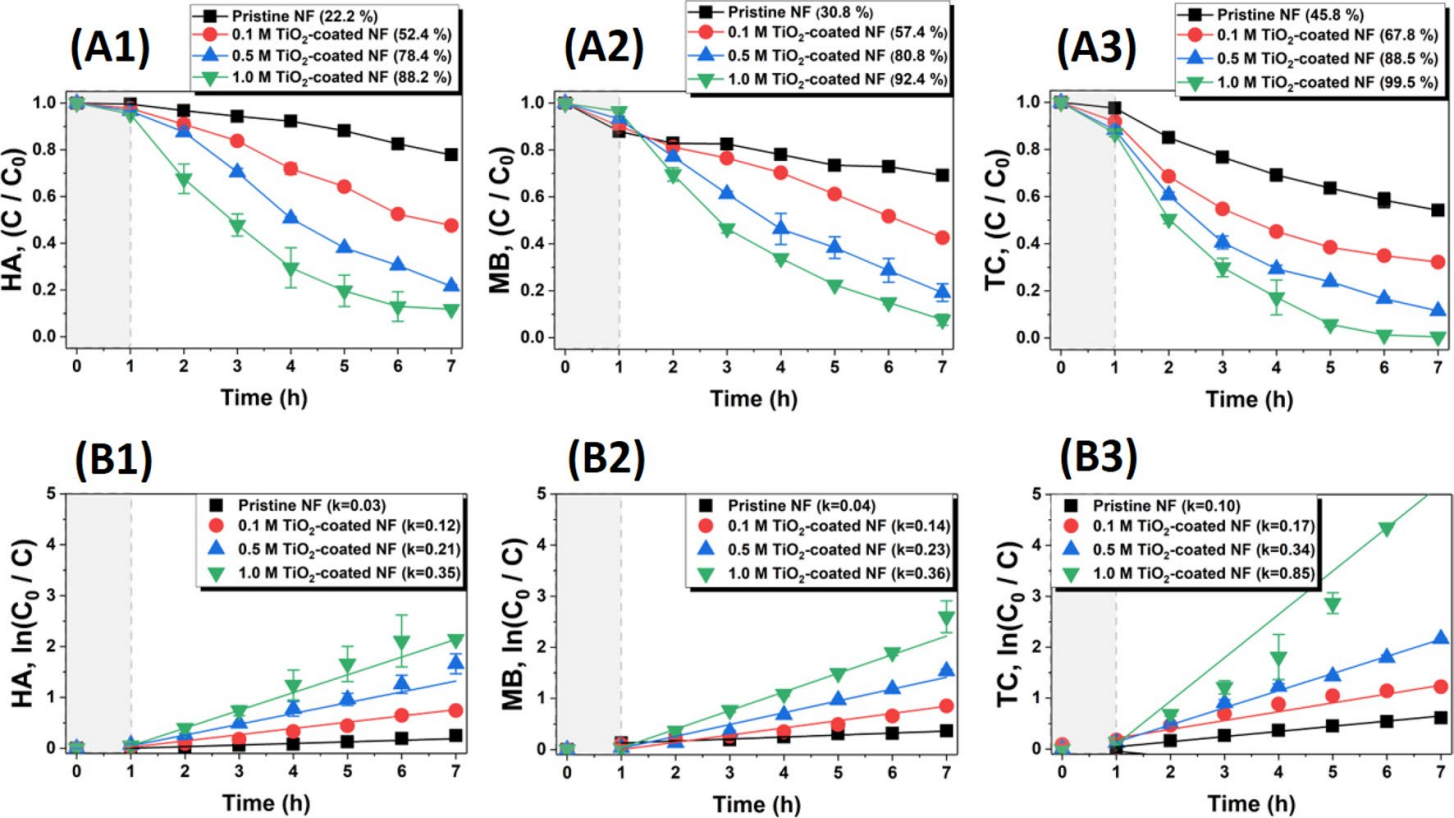
Adsorption/photocatalytic degradation of (**A1**) HA, (**A2**) MB, and (**A3**) TC and apparent reaction rate of (**B1**) HA, (**B2**) MB, and (**B3**) TC by YSZ/silica (pristine) and TiO_2_-coated YSZ/silica NF under UV-light irradiation.

The photocatalytic degradation of TiO_2_-coated YSZ/silica NF was characterized by selecting HA, MB, and TC, which represent natural organic matter, organic dyes, and antibiotics, respectively. In particular, the same pollutant concentration (20 mg/L) was adopted to compare the differences in photocatalytic degradation behavior between pollutants. The pollutant concentrations are likely to be varied for research articles, and the variations are very broad. They are mostly distributed in the range of 5 to 10 mg/L, but a relatively high concentration of 20 mg/L was used herein to demonstrate the great photocatalytic degradation performance of TiO_2_-coated YSZ/silica NF^61,62^. That is, we endeavored to accomplish the excellence of our study by using a relatively higher pollutant concentration than the commonly used pollutant concentration. Figure 7 shows the variation in the adsorption/photocatalytic degradation efficiency and the apparent reaction rate of HA, MB, and TC with UV irradiation time of the YSZ/silica NF and TiO_2_-coated YSZ/silica NF membranes. As shown in Fig. 7A1, the adsorption/degradation process of HA with the YSZ/silica NF membrane takes a total of 7 h (adsorption: 1 h, degradation: 6 h), and the entire efficiency accounts 22.2% (adsorption: 0.5%, degradation: 21.7%). The adsorption/degradation efficiency of 0.1 M, 0.5 M, and 1.0 M TiO_2_-coated YSZ/silica NF membranes were 52.4% ± 0.2% (adsorption: 2.2%, degradation: 50.2%), 78.4% ± 0.8% (adsorption: 3.6%, degradation: 74.8%), and 88.2% ± 0.3% (adsorption: 4.6%, degradation: 83.6%), respectively. In Fig. 7B1, we calculated the apparent reaction rate constant (k) of YSZ/silica NF and the TiO_2_-coated YSZ/silica NF membranes using the Langmuir–Hinshelwood rate equation^34,35^, which gave the k of YSZ/silica NF membrane for HA as 0.03 h^−1^, and the k of 0.1 M, 0.5 M, and 1.0 M TiO_2_-coated YSZ/silica NF membranes were 0.12 h^−1^, 0.21 h^−1^, and 0.35 h^−1^, respectively. The adsorption/degradation efficiency and apparent reaction rate of HA increased with an increase in the TiO_2_ content. We observed similar results for the adsorption/degradation efficiency and apparent reaction rate of MB and TC, as shown in Figs. (A2), (A3), (B2), and (B3). The difference in adsorption/degradation efficiency and the apparent reaction rate of 1.0 M TiO_2_-coated NF membrane was HA [88.2% ± 0.3% (adsorption: 4.6%, degradation: 83.6%), k = 0.35 h^−1^], MB [92.4% ± 2.3% (adsorption: 3.1%, degradation: 89.3%), k = 0.36 h^−1^], and TC [99.5% ± 0.1% (adsorption: 12.1%, degradation: 87.4%), k = 0.85 h^−1^]. As a result, the order of the adsorption/degradation pattern of 1.0 M TiO_2_-coated NF membrane is HA < MB < TC. This was caused by the difference in the (1) adsorption rate, (2) bond dissociation energy (BDE), and (3) molecular weight (MW) of each pollutant. In general, the interfacial interaction between pollutants and TiO_2_ most influences the photocatalytic degradation capability^63^. That is, the lifetime of OH radicals due to the photocatalytic activity of TiO_2_ is very short (~ ns), so that only the pollutants adsorbed on the TiO_2_ coating layer can be degraded^31^. As shown in Fig. 7, the adsorption rate for each contaminant was HA (4.6%), MB (3.1%), and TC (12.1%) (MB < HA < TC). The adsorption rates varied because the isoelectric point (IEP) was different for each pollutant. Usually, the anatase TiO_2_ has an IEP of 7.0, the IEP of HA and TC were 4.7, 4.8, respectively, and MB is a cationic dye^61,64–67^. The pH of each pollutant solution was adjusted to 6.5, and the surface charge of TiO_2_ was positive, while HA and TC were negatively charged, and MB was positively charged. As MB has the same surface charge as TiO_2_, it has the smallest adsorption rate due to electrostatic repulsion. HA and TC, which have similar IEPs, have an opposite surface charge from TiO_2_, so their adsorption rates are higher than that of MB due to electrostatic attraction. However, the adsorption rate of TC is almost 3 times higher than that of HA, this is because TC is hydrophilic^68–71^. In general, since common metal oxide ceramics are hydrophilic, the hydrophilic TC had a higher adsorption rate than the hydrophobic HA and MB^71,72^. Secondly, each pollutant has different functional groups that are collapsed by OH radical attack. The chemical structure of the pollutants affects photocatalytic degradation behavior, and in particular, the appropriate photocatalytic materials should be selected according to the functional groups. In previous studies^73–77^, the functional group cleaved by OH radicals in HA and TC is C = C (BDE: 618.3 ± 15.4 kJ/mol), while that in MB is C = S (BDE: 548.9 kJ/mol). This order of BDE of MB < HA = TC indicates that MB can be better cleaved by OH radicals than HA and TC. Finally, the MW of each pollutant is different. The MW of HA is over 10,000 g/mol, MB is 319.85 g/mol, and TC is 480.90 g/mol (MB < TC ≪ HA). Taken together, TC has the highest adsorption/degradation efficiency, because TC has the highest adsorption rate (12.1%) and relatively smaller MW (480.90 g/mol). MB has the second highest adsorption/degradation efficiency, because the lowest BDE (548.9 kJ/mol) and MW (319.85 g/mol). HA has the least adsorption/degradation efficiency, because relatively smaller adsorption rate (4.6%) and the largest MW (10,000 g/mol).

To compare the adsorption/degradation efficiency and the apparent reaction rate of 1.0 M TiO_2_-coated YSZ/silica NF membrane and TiO_2_-NPs, we measured adsorption/degradation efficiency and the apparent reaction rate using 5 mg of TiO_2_-NPs, as shown in Fig. S3. The adsorption/degradation efficiency and the apparent reaction of the 5 mg TiO_2_-NPs was HA [65.4% ± 0.9% (adsorption: 0.8%, degradation: 64.6%), k = 0.17 h^−1^], MB [67.0% ± 1.1% (adsorption: 4.8%, degradation: 62.2%), k = 0.19 h^−1^], and TC [98.4% ± 0.1% (adsorption: 2.2%, degradation: 96.2%), k = 0.69 h^−1^), respectively. It was lower than the adsorption/degradation efficiency and the apparent reaction of 1.0 M TiO_2_-coated YSZ/silica NF membrane. This is because the dispersion of TiO_2_-NPs varies depending on the shape of the container, and the distance and position from the UV light source are not constant. Moreover, the TiO_2_-NPs having high surface energy form agglomerates which can reduce the SSA of the TiO_2_-NPs and slowed the reaction of TiO_2_-NPs with UV light. As a result, it was verified that the TiO_2_-coated YSZ/silica NF membrane had higher adsorption/degradation efficiency than TiO_2_-NPs and can effectively photodegrade pollutants from different sources such as HA, MB, and TC.

**Figure 8:**
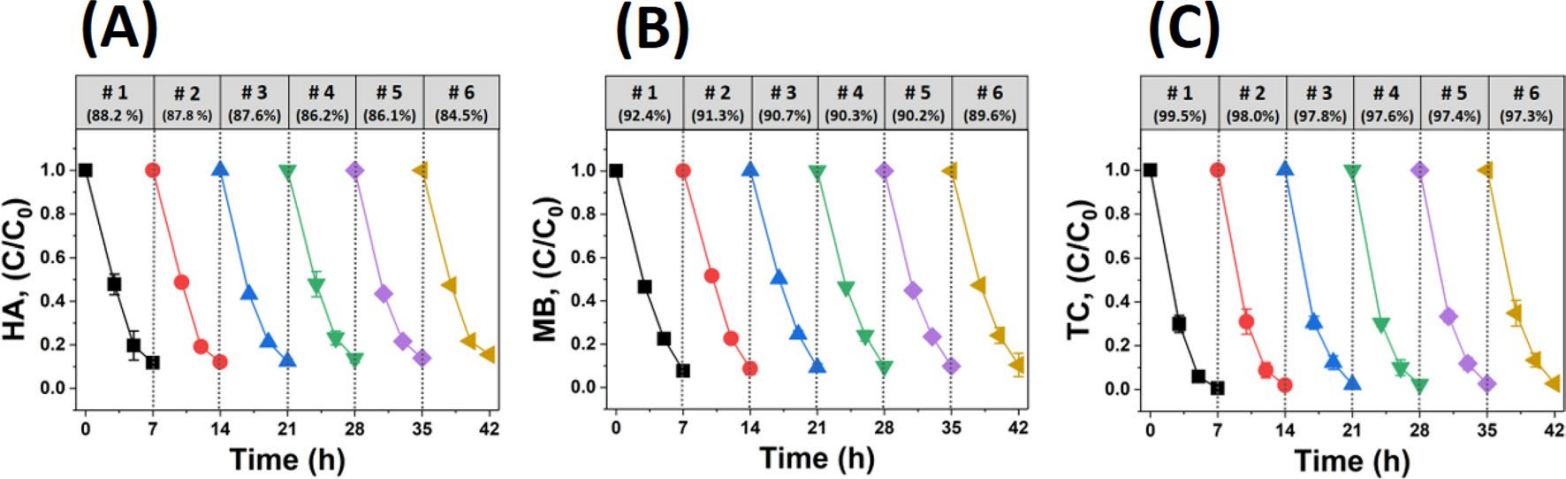
Recycling of (**A**) HA, (**B**) MB, and (**C**) TC by 1.0 M TiO_2_-coated YSZ/silica NF under UV-light irradiation.

Figure 8 shows the recycling test for HA, MB, and TC using 1.0 M TiO_2_-coated YSZ/silica NF membrane. The adsorption/degradation efficiencies of HA, MB, and TC tend to reduce during a total of six recycling tests. For example, HA decreased by 3.7% (1st cycle:88.2%, 6th cycle: 84.5%), MB by 2.8% (1st cycle: 92.4%, 6th cycle: 89.6%), and TC by 2.2% (1st cycle: 99.5%, 6th cycle: 97.3%). The reason is that the amount of HA, MB, and TC remaining after adsorption and photocatalytic degradation influenced the subsequent recycling test, as shown in Fig. 7. Teixeira et al.^78^ experimented on the recycling of Fe_3_O_4_/SiO_2_/TiO_2_ NPs using MB under UV irradiation for 90 min at each cycle, and they observed a decrease of approximately 14% (1st cycle: 95%, 5th cycle: 81%). In addition, Y. Shi et al.^19^ conducted a study on the recycling of palygorskite-supported Cu_2_O-TiO_2_ composite using TC under Xe lamp irradiation for 240 min at each cycle, and they observed a reduction by approximately 7.57% (1st cycle: 81.45%, 3rd cycle: 73.88%). The main reason for the decrease in adsorption/degradation efficiency in most of the recycling tests are as follows. In each cycle, photocatalytic nanomaterials are more likely to be lost in the recovery process such as filtering and centrifugation, which directly causes the adsorption/degradation efficiency to decrease^34,35^. Meanwhile, in order to minimize photocatalyst loss and prevent secondary environmental pollution, we developed a sol–gel process to tightly secure TiO_2_ on the surface of YSZ/silica NF membrane. Even if the photocatalytic recycling test was carried out 6 times in a row for 7 h each time, the decrease in adsorption/degradation efficiency is minimal. Such an excellent reusability is one of the greatest advantages of our study.

## Conclusions

In this study, TiO_2_-coated YSZ/silica NF was successfully developed by forming a TiO_2_ coating layer on the surface of YSZ/silica NF. This study mainly considered two aspects: (1) the ability to simultaneously perform separation and degradation and (2) improving the efficiency of photocatalytic degradation and imparting self-cleaning functionality. To achieve these goals, the TTIP concentration was controlled accordingly during TiO_2_ coating process. As the TTIP concentration increased, the TiO_2_ coating layer of the YSZ/silica NF raised. The increasing TiO_2_ coating layer reduced the pore size of the membrane, and thus the separation ability of the TiO_2_-coated YSZ/silica NF membrane was improved. Moreover, the TiO_2_ coating layer formed was able to efficiently degrade various pollutants with excellent adsorption/photocatalytic degradation. To verify the stability of the formed TiO_2_ coating layer, recycling tests were carried out, which confirmed the stability and reusability of TiO_2_-coated YSZ/silica NF membranes. In conclusion, we succeeded in developing a stable TiO_2_-coated YSZ/silica NF membrane capable of simultaneously separating and photocatalytically degrading HA, MB, and TC, which is expected to play an important role in water purification.

## Materials and methods

### Fabrication of TiO_2_-coated YSZ/silica NF

An electrospinning solution containing 1.6 M zirconium (IV) propoxide solution (Sigma-Aldrich, USA), 8 mol% yttrium (III) nitrate hexahydrate (Sigma-Aldrich, USA), 30 mol% tetraethyl orthosilicate (Sigma-Aldrich, USA), 10 wt% polyvinylpyrrolidone (Sigma-Aldrich, USA), N,N-dimethylformamide (Sigma-Aldrich, USA), and acetic acid (Sigma-Aldrich, USA) was prepared for the fabrication of YSZ/silica NF. The solution was stirred at 30 ℃ until it was clear. Electrospinning of YSZ/silica NF was conducted in a closed chamber system (NanoNC, Republic of Korea) under a controlled relative humidity (40% ± 5%) and temperature (25 ± 2 ℃). The electrospinning solution was placed in a plastic syringe and then ejected through an electrically conducting stainless-steel nozzle (23 G) at a feed rate of 1 ml/h. The applied voltage was 20 kV. The distance between the nozzle tip and the collector was set to 10 cm. The as-spun YSZ/silica nanofibers were dried in a vacuum oven at 30 ℃ for 5 h and then calcined at 800 ℃ for 2 h at a heating rate of 5 ℃/min in air. For TiO_2_ coating on the YSZ/silica NF, a TiO_2_ sol was prepared with 0.1 M, 0.5 M and 1.0 M titanium tetraisopropoxide (TTIP, Sigma-Aldrich, USA) as the TiO_2_ precursor, acetylacetone (Sigma-Aldrich, USA), 2-propanol (Samchun Chemicals, Republic of Korea), acetic acid (Sigma-Aldrich, USA), and deionized (DI) water, which were stirred at 60 ℃ for 5 h. The YSZ/silica NF were submerged in the TiO_2_ sol for 5 min under vacuum and then rinsed with 2-propanol in triplicate. The TiO_2_-coated YSZ/silica NF were dried overnight in ambient conditions and calcined at 500 ℃ for 1 h at a heating rate of 5 ℃/min in air. Finally, the TiO_2_-coated YSZ/silica NF were evaluated with respect to their physicochemical properties, membrane performance, and photocatalytic degradation.

### Characterization of TiO_2_-coated YSZ/silica NF

The surface morphology was characterized using FE-SEM (JSM-6700, JEOL, Japan) at an accelerating voltage of 5 kV and a magnification of 20,000×. HR-TEM (JEM2100F, JEOL, Japan) was also used for morphological and elemental analysis at an accelerating voltage of 200 kV and a magnification of 150,000× along with EDS (Aztec, OXFORD Instruments, UK). All electron microscope micrographs were analyzed using an image processing software (ImageJ, National Institutes Health, USA) to calculate the average fiber diameter (from more than 30 randomly selected ceramic nanofibers). The crystalline structure was analyzed with XRD (D/Max 2500 V/PC, Rigaku Corporation, Japan) using Cu kα radiation (λ = 1.5406 Å) at 40 kV and 200 mA under the scanning speed of 4°/min and a scan range (2θ) from 10 to 90°. The grain size was calculated using Scherrer’s equation^11,79^. The SSA was characterized using the BET (Autosorb-iQ, Quantachrome Instrument, USA) analysis with nitrogen as the adsorbent. TGA and DSC (SDT-Q600, TA Instruments, UK) were conducted within a temperature range of 20 to 900 ℃ at a heating rate of 10 ℃/min in an air atmosphere. The mean pore size and the largest pore size were analyzed using CFP (1200-AEL, Porous Materials, USA). The mean pore size was mainly associated with the rejection (or separation) capacity of the nanofibrous membranes. The largest pore size is more relevant to the air/water permeability (or transport property) of the nanofibrous membranes^14,80^.

### Membrane performance and photocatalytic degradation of TiO_2_-coated YSZ/silica NF

The gas permeability was tested using CFP (1200-AEL, Porous Materials, USA). The measurement was carried out at a pressure of 1.0 bar with air. The pure water permeability was determined using a cross-flow membrane testing system (PHILOS, Republic of Korea). The measurement was conducted at a transmembrane pressure (TMP) of 0.15 bar with a fluid velocity of 2.5 L/min. The rejection rate (%) was analyzed using 0.5 μm polymeric particles (Polysciences, USA). The solution containing polymeric particles was prepared by diluting it with DI water to a concentration of 250 ppm. The contents of feeding concentration (*C*_*f*_) and that of the permeate concentration (*C*_*p*_) were measured using ultraviolet–visible (UV–vis) spectroscopy (UV5, Mettler-Toledo GmbH, USA) at a wavelength of 600 nm. The rejection rate (%) was calculated using the following Eq. (1):

1 $$\text{Rejection \;rate }\left(\text{\%}\right)=\left(1-\frac{{C}_{p}}{{C}_{f}} \right) \times 100$$

The adsorption/photocatalytic degradation of the TiO_2_-coated YSZ/silica NF was evaluated using three different pollutants containing (1) HA (natural organic matter, Sigma-Aldrich, USA), (2) MB (organic dye, Sigma-Aldrich, USA) and (3) TC (antibiotic, Sigma-Aldrich, USA). The schematic diagram of adsorption/photocatalytic degradation process is described in Fig. S2. The TiO_2_-coated YSZ/silica NF membranes (50 mm in diameter) were completely submerged in the pollutant solutions (20 mg/L, pH 6.5) and left in a dark condition for 1 h to reach the adsorption equilibrium. The photocatalytic degradation was initiated using UV irradiation (30 W, 365 nm, Vilber Lourmat, France) for 6 h while the pollutant solutions were constantly shaken to ensure proper mixing. Aliquots were taken at regular time intervals (1 h) to measure the absorbance of pollutant solutions using UV–vis spectroscopy (UV5, Mettler-Toledo GmbH, USA). The specific wavelengths of 254 nm, 663 nm, and 356 nm were adopted to determine the HA, MB, and TC contents, respectively. The rate constant for the photodegradation reaction was determined using Eq. (2):

2 $$\text{ln}\left({C}_{0}/C\right)=\text{kt}$$

where, *C*_0_ and *C* are the concentrations at initial and reaction time t, respectively; k is the apparent reaction rate constant (i.e. pseudo-first-order rate constant). Furthermore, a recycling test of 1.0 M TiO_2_-coated YSZ/silica NF membrane was conducted to determine its reusability following the same way as the adsorption/degradation test described above. It was carried out 6 times in a row and reused after washing (with DI water for 1 h) and drying (in a 50 ℃ oven for 4 h) every cycle.

## Supplementary information

Supplementary Information.

## Abbreviations

k: Apparent reaction rate constant
BDE: Bond dissociation energy
BET: Brunauer–Emmett–Teller
CFP: Capillary flow porometry
DI: Deionized
DSC: Differential scanning calorimetry
EDS: Energy dispersive spectrometry
C_f_: Feeding concentration
FE-SEM: Field-emission scanning electron microscopy
HR-TEM: High-resolution transmission electron microscopy
HA: Humic acid
IEP: Isoelectric point
MB: Methylene blue
MF: Microfiltration
MW: Molecular weight
NPs: Nanoparticles
C_p_: Permeate concentration
STEM: Scanning transmission electron microscope
SSA: Specific surface area
TC: Tetracycline
TGA: Thermal gravimetric analysis
TTIP: Titanium tetraisopropoxide
TMP: Transmembrane pressure
UF: Ultrafiltration
UV–vis: Ultraviolet–visible
XRD: X-ray diffractometer
YSZ/silica NF: Yttria-stabilized zirconia/silica nanofiber
