# Supplementary material for: Development of TiO2-coated YSZ/silica nanofiber membranes with excellent photocatalytic degradation ability for water purification
Source: Sci Rep. 2020 Oct 20;10:17811. doi: 10.1038/s41598-020-74637-1 (PMC7576826; doi:10.1038/s41598-020-74637-1)
Supplement: Supplementary file 1 — Supplementary Information. [file 41598_2020_74637_MOESM1_ESM.docx]

**Development of TiO_2_-coated YSZ/Silica Nanofiber Membranes**

**with Excellent Photocatalytic Degradation Ability for Water Purification**

Jin Young Huh ^a,1^, Jongman Lee ^a,b,1*^, Syed Zaighum Abbas Bukhari ^a^,

Jang-Hoon Ha ^a^, In-Hyuck Song ^a,b^

^a^ Powder & Ceramics Division, Korea Institute of Materials Science (KIMS),

797 Changwondaero, Seongsangu, Changwon 51508, Republic of Korea

^b^ Department of Advanced Materials Engineering, University of Science & Technology (UST), 797 Changwondaero, Seongsangu, Changwon 51508, Republic of Korea

*Corresponding author: Jongman Lee

^1^Authors that equally contributed.

Powder & Ceramics Division, Korea Institute of Materials Science (KIMS),

797 Changwondaero, Seongsangu, Changwon 51508, Republic of Korea

Tel.: +82 55 280 3292, Fax: +82 55 280 3289

E-mail address: [jmlee@kims.re.kr](mailto:jmlee@kims.re.kr) (J. Lee)


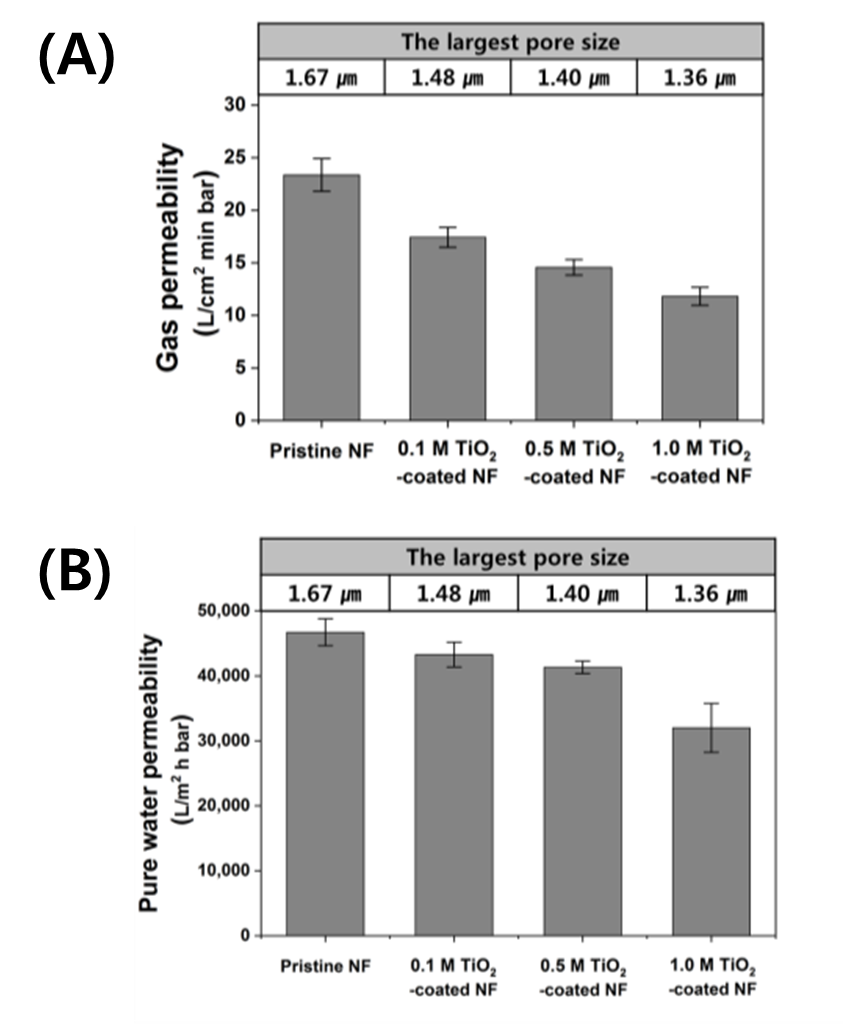


Fig. S1. (A) Gas permeability and (B) pure water permeability of YSZ/silica (pristine) and TiO_2_-coated YSZ/silica NF.


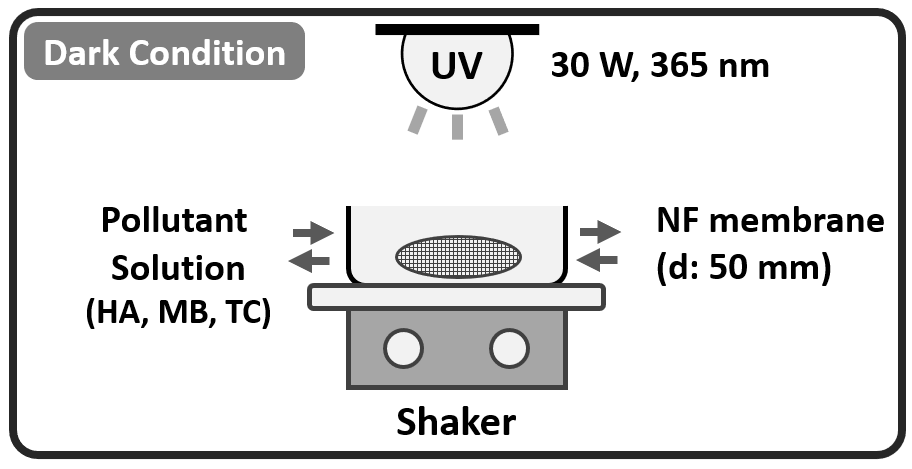


Fig. S2. Schematic diagram of adsorption/photocatalytic degradation process consisting of a UV source (30 W, 365 nm), a pollutant solution (HA, MB, and TC), a shaker, and TiO_2_-coated YSZ/silica NF membrane (50 mm in diameter).


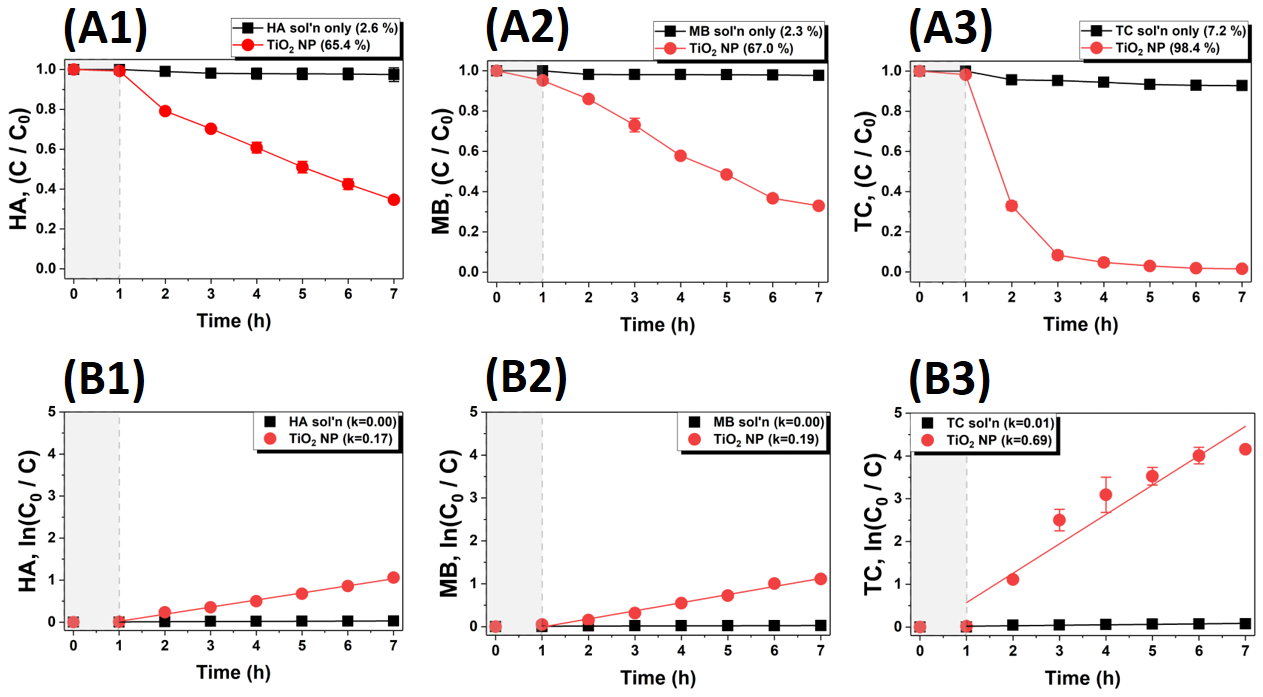


Fig. S3. Adsorption/photocatalytic degradation of (A1) HA, (A2) MB, and (A3) TC and apparent reaction rate of (B1) HA, (B2) MB, and (B3) TC by TiO_2_ NPs (5 mg) and a pollutant solution only under UV-light irradiation.
